# Histological Research and Phytochemical Characterization of *Capsella bursa-pastoris* Medik. from Bihor County, Romania

**DOI:** 10.3390/life15010067

**Published:** 2025-01-08

**Authors:** Sorina-Georgiana Onea (Minz), Annamaria Pallag, Cristina Burlou-Nagy (Fati), Tünde Jurca, Laura Gratiela Vicaș, Marian Eleonora, Neli Kinga Olah, Rita Kiss, Bianca Pașca

**Affiliations:** 1 Doctoral School of Biomedical Sciences, Faculty of Medicine and Pharmacy, University of Oradea, 410073 Oradea, Romania; onea.sorinageorgiana@student.uoradea.ro (S.-G.O.); burlounagy.cristina@student.uoradea.ro (C.B.-N.); tjurca@uoradea.ro (T.J.); emarian@uoradea.ro (M.E.); 2 Department of Pharmacy, Faculty of Medicine and Pharmacy, University of Oradea, 410073 Oradea, Romania; lvicas@uoradea.ro (L.G.V.); bpasca@uoradea.ro (B.P.); 3 Departament of Pharmaceutical Chemistry, Faculty of Pgarmacy, Vasile Goldis Western University of Arad, 310414 Arad, Romania; neliolah@yahoo.com; 4 PlantExtrakt Ltd., 407059 Cluj Napoca, Romania; 5 Department of Pharmacology and Pharmacotherapy, Faculty of Medicine, University of Debrecen, Nagyerdei krt. 98, H-4032 Debrecen, Hungary; kiss.rita@med.unideb.hu

**Keywords:** histology, anthocyanin, polyphenols, flavonoids, antioxidant capacity, HPLC method

## Abstract

*Capsella bursa-pastoris* Medik. (CBP) is a species with antibacterial, anti-inflammatory, antioxidant, anticancer, and hepatoprotective effects. We have chosen to study this species because, although it is a common plant with a distinctive fruit appearance, its effects are not fully understood. The aim of this study was to characterize the histoanatomy of the vegetative, reproductive organs and to characterize CBP extracts in terms of bioactive compounds and its antioxidant capacity. This study investigated the quantitative chemical composition of this species using the HPLC method, revealing the total content in polyphenols, flavonoids, and anthocyanins, and investigated the antioxidant potential through fluorescence recovery after photobleaching (FRAP assay), cupric ion (Cu^2+^) reduction, (CUPRAC assay), and a free radical scavenging method (DPPH). Our results show that CBP is a rich source of flavonoids, mainly from the extract obtained from the fruits; it has an antioxidant capacity, with the highest values being obtained from mature flowers and ripe fruits. Of the active principles, the highest amounts, according to HPLC determinations, were obtained in flowers and are represented by hyperoside. Thus, we can recommend the studied species for phytopharmaceutical preparations.

## 1. Introduction

Plants have been a natural resource of medication for a long time. Our continued use of herbs in today’s world highlights their beneficial effects. This acts as an encouragement for us to study the world of herbs further [1]. *Capsella bursa-pastoris* Medik. (CBP) is native to different parts of the world, including Eurasia and North Africa. It currently has a wide distribution worldwide, avoiding only lowland, tropical areas. In tropical Africa, it can be found at higher altitudes [2]. This plant can be encountered from the lowlands to the subalpine, extensively covering pastures, orchards, various crops, and even roadsides and potholes. We have chosen CBP, shepherd’s purse, as the subject of our research since it is a common plant with a distinctive fruit look, but its therapeutic effects are not fully comprehended. Evidence shows that extracts and certain components from CBP, a member of the Brassicaceae family, have antibacterial, anti-inflammatory, antioxidant, anticancer properties, and elicit smooth muscle contraction, infertility, and cardiovascular, sedative, hepatoprotective, and acetylcholinesterase inhibitor effects [3,4,5,6,7]. Because of their nutritional value, species from the Brassicaceae family are considered valuable food resources. In addition, due to the chemical composition of Brassicaceae species, whole plants or individual organs can be used as raw materials for a variety of uses [6,8]. *C. bursa-pastoris* Medik. is the world’s second most abundant wild plant, growing on cultivated land, along roadsides and in meadows [9]. Although it is native to Eastern Europe and Asia Minor, it has naturalized and is considered a common weed in many regions of the world, especially in areas with low temperatures [9,10]. It is an annual herbaceous plant, with a tap root that grows to a height of 10–40 cm. The basal leaves, which are oblong-lanceolate, are grouped in a rosette. The erect and branching stem develops from the rosette’s middle. The sessile stem leaves become smaller as they get closer to the top. The flowers are grouped in clusters of type 4 with white petals. The fruits are obcordate silicates that are triangular [11]. *Bursae pastoris* herba, the aerial part of the plant collected after flowering, is destinated for therapeutic uses. *C. bursa-pastoris* Medik. has an excellent nutritional value due to its bioactive compounds, such as phytosterols, phenols, flavonoids, fatty acids, organic acids, peptides, and amino acids, in addition to its pharmacological profile. Previous research discovered that the shepherd’s purse included a wide spectrum of compounds, including biogenic amines, resins, tannins, flavones, trace alkaloids, and mineral salts [12,13,14,15,16]. Sulforaphane is an isothiocyanate that has gained a lot of interest for its powerful chemo-preventive effect [17]. Sulforaphane levels are high in herbs that belong to the Brassicaceae family; it not only prevents chemically generated tumors in animal models, but also inhibits the development of existing tumors [18,19]. Because of their phenolic compounds, Brassicaceae’s dietary intake aims to provide health advantages, such as their ability to prevent cancer, prevent aggregation, and activate detoxifying enzymes. *CBP* is often encountered in specialized studies for its effects on the uterus, being used in menorrhagia and metrorrhagia [17]. A study has shown that 96% hydroalcoholic extracts (which were mixed with starch powder and then filled into size zero capsules, each capsule containing 320 mg of the shepherd’s purse extract equal to 2.5 g of the herb) from CBP capsules given after 12 h were more effective in stopping heavy menstrual bleeding than 500 mg of mefenamic acid given after 8 h [20]. It is also being studied for its infertility effects, with studies showing that CBP, dried and ground, added in a certain proportion to the diet of male and female mice, can cause temporary infertility [17]. It has also been studied for its cardiovascular effects and anti-cancer effects, all of which have only been studied in the internal part of the body. Because CBP has a high antioxidant capacity and a variety of phenolic compounds with anti-inflammatory capacity, in our opinion, CBP can be used to treat a variety of dermatologic conditions, an example being skin ulcers [21].

The aim of this research was the histological characterization of different organs and the characterization of *C. bursa-pastoris* Medik. extracts in terms of bioactive compound, polyphenol and flavonoid content; antioxidant capacity; and quantitative determinations of compounds by HPLC. These determinations will be used to determine the optimal concentrations to introduce the extracts into a subsequent pharmaceutical product [22].

## 2. Materials and Methods

### 2.1. Plant Material

CBP samples used in this study, originating from the spontaneous flora, were been cautiously harvested in 2023 (between April and June), from unpolluted regions in the Oradea area (Bihor County, Romania), the type of soil being preluvosoil, with a clayey texture, wealthy in carbonates. A sample of the species has been preserved inside the herbarium of the Faculty of Medicine and Pharmacy Oradea, Romania, registered in NYBG Steere Herbarium, UOP 05136. Four exclusive organs—root, leaf, flowers, and fruits—were collected.

From the collected organs, we made microscopic sections, but in order to perform the chemical analysis, they were dried at an average temperature of 40 °C, for 96 h.

### 2.2. Microscope Analysis

Microscope slide preparations were made by different methods from fresh plant material. An optical microscope, Optika B383LI (Ponteranica, Italy), was used, with 10×, 20× and 40× objectives. Cross sections of different organs of CBP, up to 1 cm in diameter, covered the entire area of the microscope field. The following dyes were used: Genevez reagent (aqueous ammonia solution of Congo red and alcoholic chrysoidine solution, prepared 1:1), and methylene blue solution, to highlight the histologic structures of CBP.

### 2.3. Phytochemical Analysis

The different plant organs were grounded to a powder. A 10% solution of each product was then prepared: 10 g of plant product was dissolved in 100 mL of methyl alcohol solvent and then sealed with parafilm. The extract was allowed to stand for 7–10 days before filtration. Filtration was conducted through a cloth.

#### 2.3.1. Determination of Total Polyphenol Content

**Figure 1:**
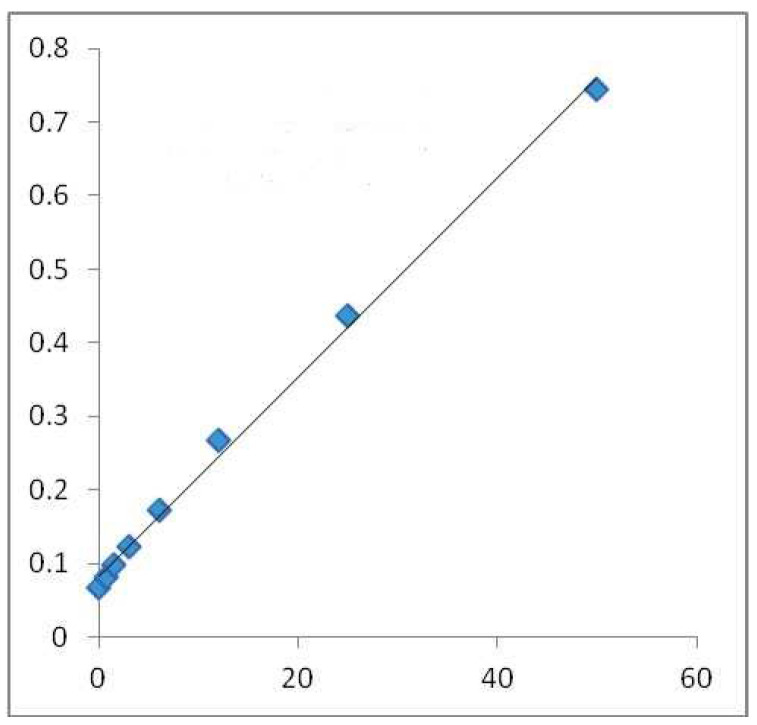
Calibration line made with gallic acid for Folin–Ciocalteu method in alcoholic medium. Absorbance 765 nm (concentration of gallic acid mg/GAE/100 g DW) where the blue squares highlight the mg/mL values used for the regression equation.

In order to reveal the total amount of phenol in the sample, the Folin–Ciocalteu technique was used. Given an alkaline medium, the examined sample’s OH groups could be assessed by applying the Folin–Ciocalteu technique (adjusted with Na_2_CO_3_). In a direct ratio to the number of OH groups present in the polyphenols, the absorbance at 765 nm wavelength rose. The extract solution (0.1 mL), which contained 1000 μg of the extract, was combined with 46 mL distilled water in a volumetric flask; 1 mL Folin–Ciocalteu (Merck Chemicals, Darnstadt, Germany) reagent was also added, and they were mixed well together by vigorously shaking the flask. The resulting mix was allowed to react for 3 min and an additional 3 mL of aqueous solution of 2% Na_2_CO_3_ was added. After the mixture was left aside for a 2 h incubation at room temperature, the absorbance of each mix was calculated at 765 nm with the Shimadzu UV-1700 Pharmaspec UV-VIS Spectrophotometer. This procedure was also applied to the standard solutions of gallic acid, which resulted in a standard line (Figure 1). The results are be expressed in mg/GAE/100 g DW [23].

#### 2.3.2. Determination of Total Flavonoids

**Figure 2:**
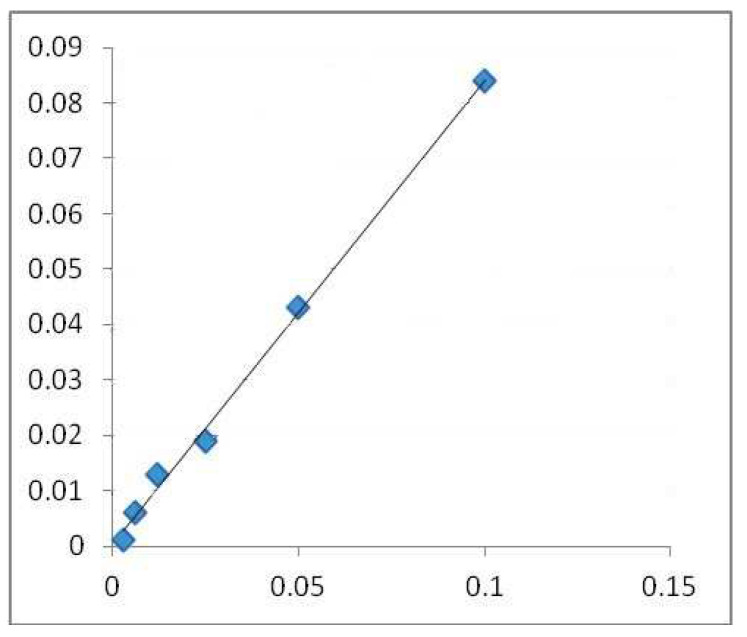
Calibration line made with quercetin in alcoholic medium (surroundings, environment). (Absorbance 510 nm (concentration of quercetin mg QE/g DW).), where the blue squares highlight the mg/mL values used for the regression equation.

A colorimetric method was used to determine the total flavonoid content [23]. A 10 mL volumetric flask was filled with a 1 mL test (containing 0.1 mg/mL of dry material) after diluting it with 4 mL of water. The first addition was 3 mL of 5% NaNO_2_ solution, followed by 0.3 mL of 10% AlCl_3_ after 5 min and 2 mL of 1M NaOH after 6 min. Distilled water was added to the flask until it reached the calibration point. Then, the solution was blended, and the Shimadzu UV-1700 Pharmaspec UV-VIS Spectrophotometer measured the solution’s absorbance at 510 nm [24,25,26]. The results are be expressed in mg QE/g DW. The calibration line (Figure 2) was created using quercetin (QE) as its standard.

#### 2.3.3. Total Anthocyanin Content Determination

In order to reveal the total anthocyanin composition, a method detailed in specific studies was used [26]. When applying this method, the specimens had to be diluted (5:95, *v*/*v*) in 1% HCl in methanol to obtain a retention of 0.200–1.000 at 530 nm, and the outcomes were expressed in mg cyanidin/100 gDW.

The determination of the total content of anthocyanins was achieved by a method based on the anthocyanins’ property of changing their color depending on the pH.

The determination of anthocyanins is based on a basic principle, that the anthocyanins show a diverse absorbance spectrum. At pH 1.0, the oxonium colored form is predominant and at pH 4.5, the cetalin form is present. This spectrophotometric procedure, based on the pH differentiation, enables the calculation of the total anthocyanins, in spite of degraded polymerized pigments or other elements that can alter the environment.

Materials: potassium chloride buffer, 0.025M, pH 1.0; sodium acetate buffer, 0.4 M, pH 4.5.

Working method: Roughly 0.15 g of each plant part was measured, which was homogenized with the aid of the Ultraturex, for 1 min at 3000 rpm, in methanol acidified with HCl 0.3%.

After this operation, the samples were centrifuged for 20 min at 5000 rpm. Thus, the supernatant was isolated, and the residue was homogenized again by centrifugation, repeating the process 3 times. The supernatants were pooled, and from them, both the anthocyanin content and the antioxidant activity were calculated. In the next steps, there were two dilutions of the samples made, one at pH 1.0 in KCL buffer and one at pH 4.5 in acetate buffer, ensuring that the absorbance of the sample at λvis-max did not surpass 1.2. The samples were then allowed to rest for 15 min, after which new measurements were conducted: the absorbance for each dilution was measured at λvis-max and 700 nm against the solvent used [26]. The absorbance of the diluted samples was then calculated as shown in the formula below:

A = (A λvis-max − A700) pH 1.0 − (A λvis-max − A700) pH 4.5(1)

For the calculation of the monomeric anthocyanin pigment in the sample, the following formula was used:

The monomeric anthocyanin pigment (mg/L) = (A × Mw × DF × 1000)/(ε × 1)(2)

where A is the absorbance calculated in the case of Equation (1), MW is the molecular mass, DF the dilution factor, and ε the molar extinction coefficient.

The content in the monomeric pigment was calculated as a function of cyanidin-3-glucosylated, which has values of MW = 449.2 and ε = 26.900.

#### 2.3.4. Antioxidant Capacity

##### Fluorescence Recovery After Photobleaching (FRAP)

When applying the FRAP method, the ferric–tripyridyl triazine complex [Fe(III)-TPTZ] is reduced by a reductant at an acidic pH. This spectrophotometric technique allows the measurement of the antioxidant capacity of the studied specimens. The stock solutions contained 270 mg of FeCl_3_-6·H_2_O dissolved in 50 mL of distilled water, 150 mg of TPTZ, 150 mL of HCl dissolved in 50 mL of distilled water, and 300 mM acetate buffer. The freshly prepared working FRAP solution was obtained by combining 5 mL FeCl_3_-6·H_2_O solution, 5 mL TPTZ solution, and 50 mL acetate buffer. In this case, Trolox was used as the standard solution and the calibration curve was set up for concentrations between 0 and 300 μM with a correlation coefficient R^2^ = 0.9956 and the regression equation y = 0.0017x + 0.0848), y representing the absorbance detected at 595 nm [27].

The results are displayed as µmol Trolox equivalent (TE)/100 µL extract [28].

##### Cupric Ion (Cu^2+^) Reducing CUPRAC Assay

The antioxidant reducing capacity of cupric ions was determined using the method offered by Karaman et al. (2010), with minor alterations [29]. The process consisted of putting 0.25 mL ethanolic solution of neocuproine (7.5 × 10^−3^ M), 0.25 mL CuCl_2_ solution (0.01 M), and 0.25 mL CH_3_COONH_4_ buffer solution (1 M) in a test tube and combining them with the plant extract. The entire amount was gently mixed and adjusted to 2 mL with additional distilled water, leaving the stoppered tubes at room temperature for 30 min. After that, the absorbance was measured at 450 nm against a reagent blank. The augmented absorbance indicated an increased reduction capacity [3,22,30,31].

##### ABTS Method

The method is based on the ability of antioxidants to reduce the cation radical (ABTS^+^, 2′-Azinobis-(3-Ethylbenzthiazolin-6-Sulfonic Acid), a green-blue chromophore that absorbs at 734 nm. In general, ABTS + is the result of the reaction, at room temperature, between ABTS solution (7 mM) and potassium persulfate (2.45 mM), protected from light for 16 h. The method is compared to the Trolox standard. After preparing the ABTS^+^ cation radical, the sample (0.1 mL) was mixed with ABTS^+^ (0.9 mL) and the absorbance was read at 734 nm, after incubation for 0.5 h. The results were expressed in µmol Trolox/mL extract. The ABTS value was obtained using the following calibration curve:

y = 629x + 98.94 (R^2^ = 0.998),

where x was the absorbance and y was the equivalent of Trolox µmol [27,31].

##### Free Radical Scavenging Method (DPPH)

The measurement of the radical scavenging activity of plant extracts against stable 2,2-diphenyl-2-picrylhydrazyl hydrate (DPPH) was carried out using a technique developed by (Brand-Williams et al., 1995), which was slightly altered [3,22,32]. Combining DPPH with an antioxidant component leads to the reduction of DPPH and the donation of hydrogen. In order to detect the color change (from intense violet to bright yellow), a UV visible light spectrophotometer was used, at 517 nm. Before measuring UV, a fresh solution of DPPH in 6 × 10^−5^ M methanol was prepared daily. The samples were kept away from light at room temperature for 15 min and the decrease in absorbance was calculated. The experiment was performed in triplicate and the radical absorbance activity was determined using the following formula:

% Inhibition = [(AB − AA)/AB] × 100

where AB = the absorption of the blank sample (t = 0 min), AA = the absorption of the test extract solution (t = 15 min) [33].

#### 2.3.5. HPLC Analyses

One gram of the samples was taken and extracted with 5 mL of methanol and then sonicated for one hour. The obtained mixture was centrifuged (15,269× *g*) for 10 min. The supernatants were then gathered, microfiltered as in the above process, and then used for HPLC/diode array detection array (DAD) analysis.

Afterwards, extracts were analyzed with a Shimadzu Nexera-I HPLC using a silica gel C18 column, Fortis C18, 150 × 2.1 mm × 3 µm system by an acidified water–acetonitrile gradient, as explained in the following steps: water was adjusted to pH 2.5, with 0.1% formic acid (A) and acetonitrile (B) used as solvents. Using a linear gradient, the start point was 80% A, which decreased to 60% within the following 5 min, to 40% by the next 10 min, then to 20% by 15 min and to 20% by the end of another 5 min. The solvent(A)’s concentration lowered to 10% and stood like this for another 5 min, the A phase was raised to 20% over the next 5 min, then progressively increased until, at the end of the 40 min analysis, it reached 80%. The entire spectral results obtained were concentrated in the range of 220–600 nm. In order to check the linearity of the detector response [31], the following standards were used: chlorogenic acid, caffeic acid, trans p-coumaric acid, ferulic acid, gallic acid, apigenin, rutosid, myricetin, myricetin, quercitrin, quercetin, luteolin, luteolin-7-glucoside, and kaempferol [34].

In Table 1, the parameters for calibration curves in different polyphenols are given.

## 3. Results

### 3.1. Histological Structure of CBP Organs 

#### 3.1.1. Histologycal Structure of *Capsella bursa-pastoris* Medik. Root

**Figure 3:**
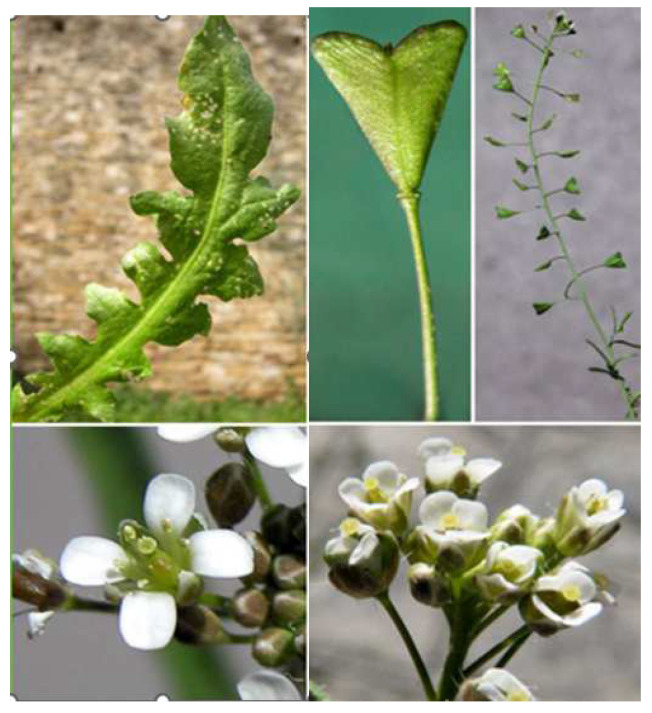
*Capsella bursa-pastoris* Medik. in the spontaneous flora of Romania.

The microscopic analysis of the cross sections through the underground organ establishes their structure, the type of vascular tissues, reserve materials, the presence or absence of crystallized inclusions, and the type of mechanical tissues. The sequence of tissues from the outside to the inside is as follows: exoderma, cortex, central cylinder, and medullary rays. The exoderma has the role of protective tissue. The cortex comprises several layers of living, parenchymal cells, with thin, cellulosic walls and intercellular spaces (Figure 3).

The central cylinder is composed by the pericycle, the vascular tissues formed by xylem and phloem, arranged alternately with medullary parenchyma.

**Figure 4:**
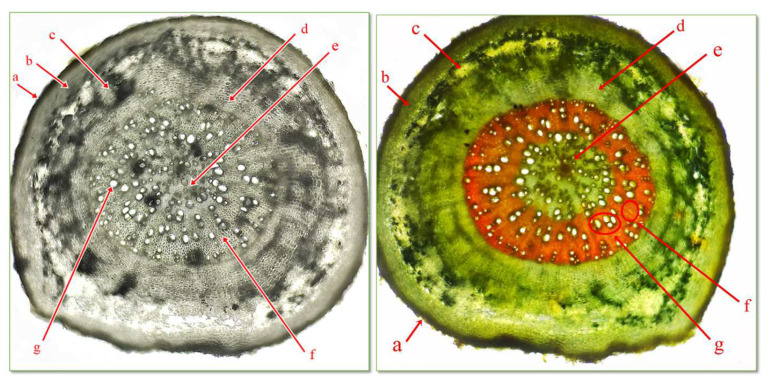
Cross section through root of *C. bursa-pastoris* Medik. (400×) (Genevez reagent staining). a = exoderma; b = primary cortex; c = primary phloem; d = secondary phloem; e = medullary parenchyma; f = primary xilem; g = secondary xylem.

In the vascular tissues, we can distinguish the primary xylem, arranged in the vicinity of the pericycle, with a small diameter, which primary formed during ontogenesis, and the secondary xylem, formed later, with a larger diameter, arranged towards the central part of the central cylinder. The primary phloem represents the youngest elements, in which are circulating organic compounds. The secondary phloem, which forms later, is arranged towards the central part. The central part of the root is occupied by the central pith composed of parenchyma cells. Medullary rays are those running from the pith to the peripheral part to the phloem (Figure 4).

#### 3.1.2. Histological Structure of *C. bursa-pastoris* Medik. Stem

**Figure 5:**
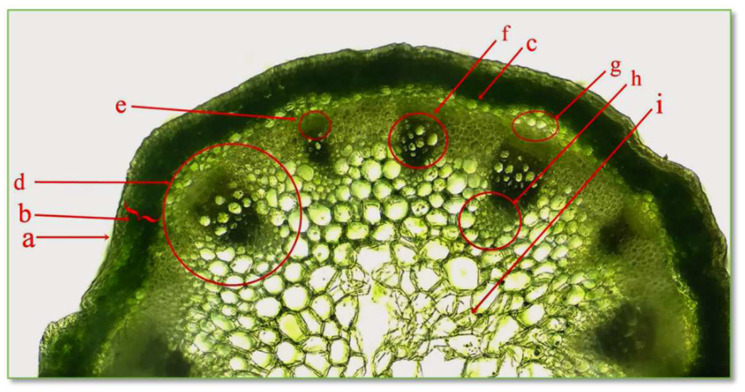
Cross section through stem of *C. bursa-pastoris* Medik. (400×). a = epidermis; b = primary cortex; c, g = pericycle; d = phloem vessel; e = vessel; f, h = xylem vessel; i = central pith.

The cross section through the stem of the *CBP* establishes the structure of the protective tissue, cortex, central cylinder, and medullary parenchyma, as seen in Figure 5.

The epidermis is the protective tissue of the stem; being composed of a single layer of living cells, closely joined together, with slightly convex external walls, the cell walls are cutinized. The cortex, also called the cortical parenchyma, is located between the epidermis and the central cylinder and is composed of numerous layers of parenchyma cells, with thin, cellulose walls. The peripheral layers of cells are represented by assimilating tissues called collenchyma, with cells that contain chloroplasts and have a role in photosynthesis. The central cylinder is formed of the pericycle, vascular fascicles, and medullary parenchyma. The pericycle is the first tissue, located immediately under the cortex, consisting of a single layer of cells with thin, cellulosic walls. The vascular tissues, xylem, and phloem are brought together in collaterally open conducting bundles, playing a role in the transport of water and nutrients. The primary xylem appears first and consists of vessel elements, and the walls are slightly lignified, allowing growth in length. The secondary xylem appears later and consists of cells with a large lumen; the walls are strongly thickened. The primary and secondary phloem consist of vessel elements without major differences between them. The medullary parenchyma is present in the central part of the stem, arranged in a rosette.

#### 3.1.3. The Structure of *C. bursa-pastoris* Medik. Leaves’ Epidermis and Protective Trichomes

**Figure 6:**
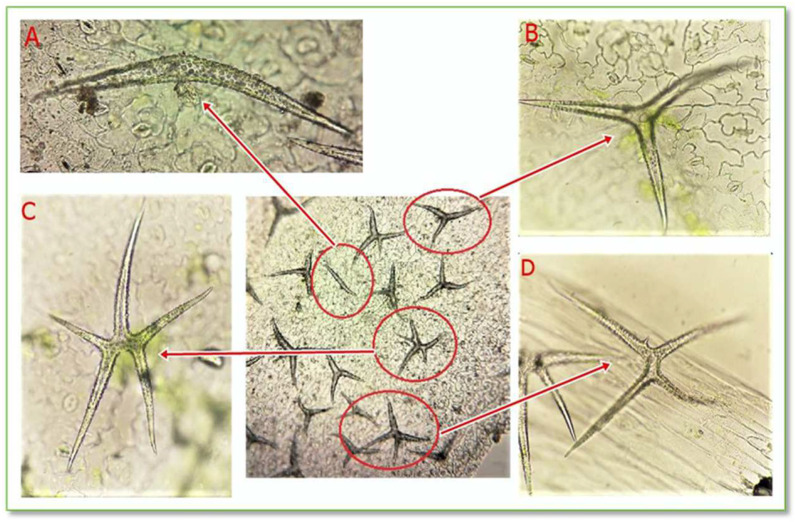
Stellate unicellular trichomes from the leaf epidermis of *C. bursa-pastoris* Medik. (100× and 400×); (**A**) unicellular trichomes with 2 branches; (**B**) unicellular trichomes with 3 branches; (**C**) unicellular trichomes with 5 branches; (**D**) unicellular trichomes with 4 branches.

**Figure 7:**
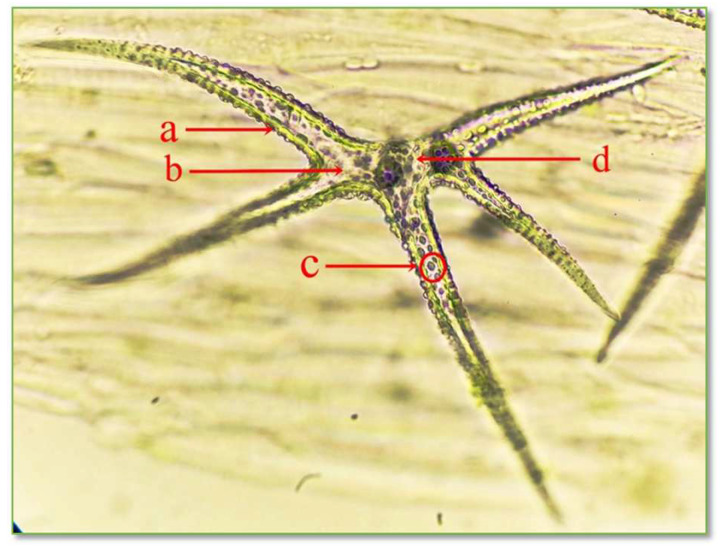
Structure of unicellular stellate protective trichomes with 5 branches on the leaf epidermis of *C. bursa-pastoris* Medik. (400×): a = cell wall; b = cytoplasm; c = ergastic inclusions; d = nucleus.

The epidermis is a protective tissue located on the surface of the leaf, having the role of sheltering it against unfavorable environmental agents. The highlighted protective trichomes on the *CBP* leaf epidermis are unicellular stellate trichomes with a variable number of branches, as seen in Figure 6 and Figure 7.

### 3.2. Phytochemical Analysis of C. bursa-pastoris Medik. Extract

The total contents of flavonoid and polyphenol comprised in the extract are different, depending on the aerial part of the plant for which they are determined, as is shown in Table 2. It can be observed that CBP fruits have the highest content in polyphenolic and flavonoid compounds. They contain 624.23 mg/GAE/100 g DW of total polyphenols and 23.14 mg QE/100 g DW of total flavonoids. In terms of anthocyanin content, it can be observed that shepherd’s milkweed flowers have a higher value, 7.1805 mg cyanidin/100 g DW.

Thus, we evaluated the antioxidant activity of the plant extract using the DPPH, ABTS, CUPRAC, and FRAP methods. The obtained results are summarized in Table 3. The determinations were made for extracts obtained from the root, flowers, leaves and fruits of the shepherd’s milkweed. Using the DPPH, CUPRAC, and FRAP methods, the results demonstrate that the flowers have a higher antioxidant activity, while using the ABTS method, it can be observed that the leaves have a higher antioxidant capacity.

#### The Results of the HPLC Analyses

**Figure 8:**
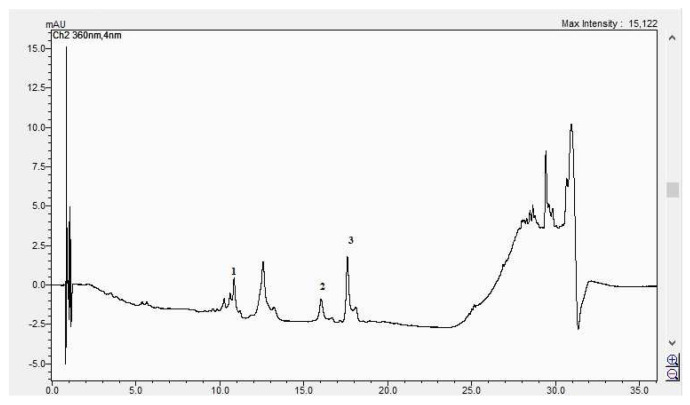
The HPLC chromatogram of the CBP root sample (1 = ferulic acid, 2 = rutosid, 3 = quercitrin).

**Figure 9:**
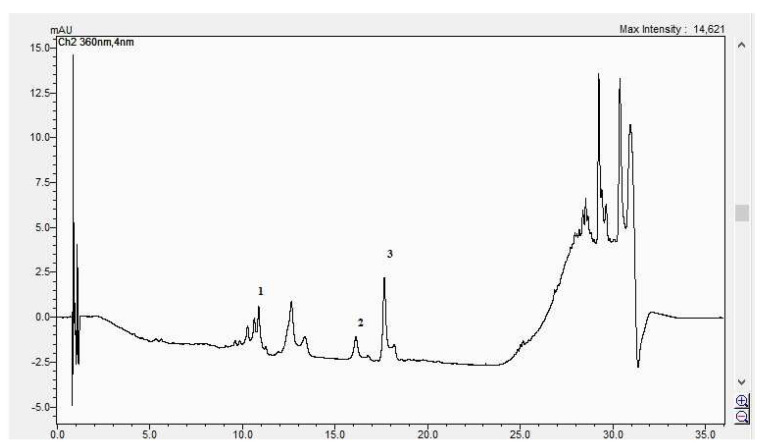
The HPLC chromatogram of the CBP leaves sample (1 = ferulic acid, 2 = rutosid, 3 = quercitrin).

**Figure 10:**
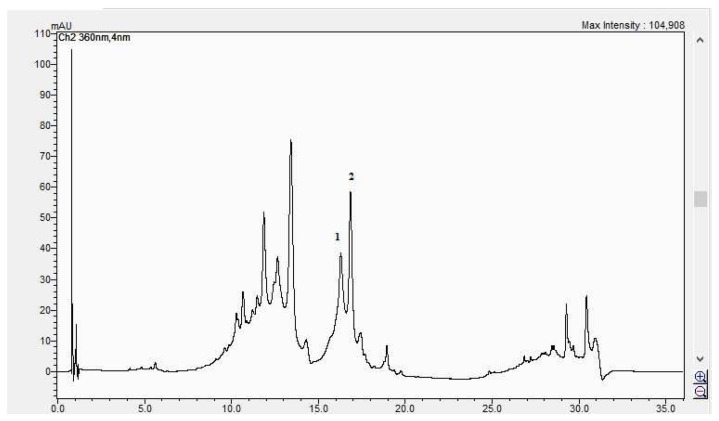
The HPLC chromatogram of the CBP flowers sample (1 = hyperosid, 2 = rutosid).

**Figure 11:**
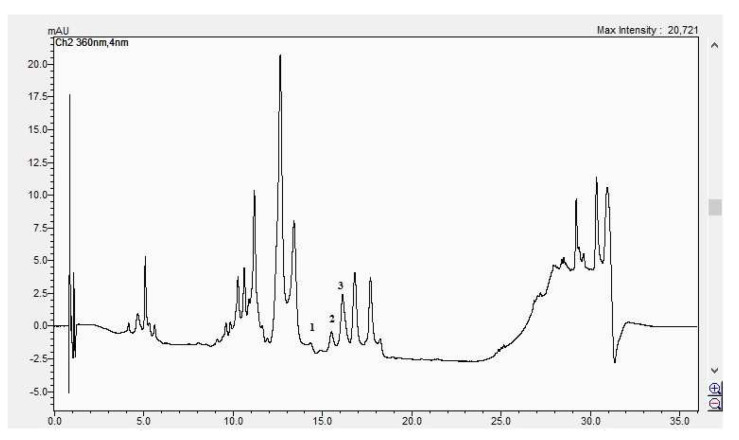
The HPLC chromatogram of the CBP fruits sample (1 = luteolin-7 *O*-glucoside, 2 = hyperosid, 3 = rutosid).

In Figure 8, Figure 9, Figure 10 and Figure 11, the HPLC chromatograms of the samples recorded at 360 nm are shown.

Table 4 contains data regarding the identification and quantification of the HPLC analysis results. The identification was carried out through the comparison of retention times and the UV-Vis spectra maximum absorption of standards and separated compounds from samples.

## 4. Discussion

Shepherd’s purse is reported to be a good source of bioactive compounds due to its content of various phytochemicals. For these reasons, this study investigated the chemical composition of this species using the HPLC method and its antioxidant activity using the following methods: FRAP, ABTS, CUPRAC, and DPPH, while also leading to the identification of the total polyphenols, flavonoids, and anthocyanins [31]. The antioxidant activity of the extract was highlighted, suggesting that it may prove of interest in regard to human health [35], particularly given the vital role of vitamin D in supporting overall well-being [36,37,38]. In another study conducted on this subject, the analysis of the total polyphenols and flavonoids in the alcoholic extract of shepherd’s purse was presented, using gallic acid as a standard. As a result, the researchers obtained 29 mg GAE/g powder. The percentage of tannin in terms of gallic acid in the *C. bursa-pastoris* plant was 4.57% [31]. Thus, differences can be observed between the results obtained by us and those obtained by other researchers, these results being influenced by environmental variables such as soil composition, temperature, precipitation, and UV intensity. Also, the amount of plant polyphenolic compounds and antioxidant capacity depends on biological factors (genotype, organ, and ontogeny) [39]. Using the CUPRAC method, *C. bursa-pastoris* Medik. has the lowest antioxidant capacity of 98.06 μmol Trolox/mL, according to a study using a 50% ethanolic extract. The researchers of the study also determined the antioxidant capacity by the FRAP method, through which we can observe low antioxidant activity [40]. Several researchers determined the antioxidant activity of *C. bursa-pastoris* Medik. by the DPPH method, obtaining an IC50 of 235.37 μg/mL and 552.01 μg/mL-1 for chloroform and methanol extract, respectively. According to that study, shepherd’s purse might provide a good supply of protein, energy, and minerals for human consumption in the form of green vegetables [41]. In another study, the identification and measurement of primary metabolites (organic acids, amino acids, and fatty acids) and secondary metabolites (phenolic compounds and sterol derivatives) from *CBP* as well as the screening of a series of biological activities were performed [28]. These include kaempferol, quercitin, kaemphferol-3-o-rutozide, tricin, oxalic acid, malic acid, and fumaric acid. [17]. Peng et al. identified by the HPLC method 24 chemical compounds, including phenolic acids and flavonoids [41]. Our study was conducted to determine the active principles found in *C. bursa-pastoris* and its antioxidant capacity. We also determined the organs of the CBP that are richer in active principles in order to introduce them in subsequent phytopharmaceutical preparations. Our determinations show that the CBP flowers represent rich sources of polyphenols (567.12 mg GAE/g DW), flavonoids (15.66 mg QE/g DW), and anthocyanins (7.1805 mg/100 g DW). Antioxidant activity has been monitored by several methods and in several plant organs. The results show that flowers have the strongest antioxidant effect. Among the active principles, the highest amounts, according to the HPLC determinations, were obtained in flowers and are represented by rutozide and hyperoside. The results obtained show some differences compared to the results of other works. The differences are probably due to the specific pedoclimatic conditions in the harvesting area in Bihor County (Romania).

## 5. Conclusions

*C. bursa-pastoris* Medik. is a plant with a diverse spectrum of chemical compounds, aiding in a wide range of pharmacological actions. Because of its efficacy and safety, *C. bursa-pastoris* Medik. has a lot of potential for the development of innovative medications to treat a variety of human diseases. The histological characterization highlighted the main morphological characters for recognizing and identifying the species. Our results from a phytochemical analysis show that *C. bursa-pastoris* Medik. is an abundant source of active principles, it is rich in flavonoids, mainly from the extract obtained from the fruits, and it has an antioxidant capacity, with the highest values being obtained from flowers and fruits. Among the active principles, the highest amounts, according to HPLC determinations, were obtained in flowers and are represented by rutozide and hyperoside. Therefore, the studied species is recommended for phytopharmaceutical preparations, to be realized by our research team.

## Figures and Tables

**Table 1:** The calibration curves of standard polyphenols.

Name	Equation	R^2^	Limit of Detection, μg/mL	Limit of Quantification, μg/mL
Apigenin	A = 42,007 × c − 218,952	0.9990	15.6	26.1
Caffeic acid	A = 87,231 × c − 239,800	0.9994	5.5	11.0
Chlorogenic acid	A = 20,718 × c − 10,584	0.9975	1.0	2.0
Ferulic acid	A = 103,386 × c − 693,158	0.9992	26.8	40.2
Hyperosid	A = 35,253 × c − 185,515	0.9979	10.5	21.0
Luteolin-7 O-glucoside	A = 41,108 × c + 415,216	0.9940	20.2	40.4
Quercitrin	A = 30,871 × c − 229,685	0.9948	22.3	37.2
Rutosid	A = 34,187 × c + 67,369	0.9985	2.0	7.9

**Table 2:** The content of polyphenols, flavonoids, and anthocyanins in the extract of *C. bursae-pastoris* Medik.

Total Bioactive Compounds	CBP Root	CBP Flowers	CBP Leaves	CBP Fruits
Content in total polyphenols (mg GAE */g DW)	231.12	567.12	344.23	634.23
Total flavonoids (mg QE **/g DW)	2.34	15.66	17.88	23.14
Anthocyanin (mg cyanidin/100 g DW)	0.00	7.1805	0.5009	5.176

* GAE: gallic acid; ** QE: quercetin.

**Table 3:** Antioxidant activity determined by the four chemical methods of the *C. bursa-pastoris* Medik.

Part of the Plant	DPPH %	CUPRAC (μmol Trolox Equivalent/mL)	ABTS (μmol Trolox Equivalent/mL)	FRAP (μmol Trolox Equivalent/100g)
CBP root	45.6	155.2	219.48	100.12
CBP flowers	87.07	1318.33	199.93	753.64
CBP leaf	83.33	361.66	286.28	614.23
CBP fruits	86.35	506.66	212.97	511.88

**Table 4:** The identification and quantification data of the HPLC analysis results.

Sample	Identified Compound Name	Retention Time, min	Maximum Absorbance, nm	Quantification, % mg/g Plant Material
Standards	Apigenin	23.3	338	
	Chlorogenic acid	6.5	326	
	Ferulic acid	10.8	322	
	Hyperosid	15.3	355	
	Luteolin-7 *O*-glucoside	14.4	349	
	Quercitrin	17.6	350	
	Rutosid	15.8	355	
CBP root	Ferulic acid	10.8	322	3.86 ± 0.045
	Quercitrin	17.6	350	8.08 ± 0.031
	Rutosid	16.0	354	3.45 ± 0.011
CBP leaves	Ferulic acid	10.9	323	3.86 ± 0.038
	Quercitrin	17.7	350	7.44 ± 0.052
	Rutosid	16.1	353	0.24 ± 0.008
CBP flowers	Hyperosid	15.2	352	80.0 ± 15.51
	Rutosid	16.1	353	110.0 ± 27.52
CBP fruits	Hyperosid	15.5	352	10.66 ± 0.065
	Luteolin -7 *O*-glucoside	14.4	348	7.30 ± 0.055
	Rutosid	16.1	353	5.77 ± 0.035

## Data Availability

The original contributions presented in the study are included in the article; further inquiries can be directed to the corresponding author.
